# Toxæmia in the Acute Specific Fevers

**Published:** 1914-07-18

**Authors:** A. Knyvett Gordon

**Affiliations:** formerly Lecturer on Infectious Diseases in the University of Manchester and Medical Superintendent of the Monsall Hospital


### TOXÆMIA IN THE ACUTE SPECIFIC FEVERS.

By A. KNYVETT GORDON, M.B. Cantab., formerly Lecturer on Infectious Diseases in the University of Manchester and Medical Superintendent of the Monsall Hospital.

In the present paper I purpose drawing attention to some points in connection with the toxæmia of the commoner acute specific fevers to which experience has led me to attach some importance. I do not propose to deal with the subject in detail, or to enter deeply into the most interesting question of its pathology: my object is mainly clinical.

Let us consider what actually happens when a patient "catches" a fever. Now, whether we are familiar with the causative agent itself, as in the case of enteric fever, or whether this is unknown, as in measles, we are certain that a parasite of some sort gains entrance into the body. For a time, which varies with the particular disease, and which we call the incubation period, nothing very definite happens. It is true that the infecting agent *may* be present in the circulating blood, but we see nothing clinically (except indefinite *malaise* in the patients who are going to be seriously ill) which we can diagnose with any degree of accuracy. During that time, however, it is supposed that toxins are being formed, but are being restrained from having much effect on the patient by the activity of the leucocytes. Why this incubation period should have a definite length in so many of the specific fevers we do not know—and I am not sure, incidentally, that we are not apt to be too certain about its definiteness—but when the patient comes to the time of invasion we get the clinical signs, which are associated with the presence of toxins in marked quantity in the circulating blood.

These signs are well known; pyrexia, headache, sometimes vomiting, thirst, prostration, and so on, and they vary to a certain extent in kind with the particular fever and in degree with the severity of the attack. Perhaps we are accustomed to lay most stress on the pyrexia, but it seems to be necessary to interpose a word of caution here. Certainly the more one sees of specific fevers the less is one inclined to be apprehensive of pyrexia *per se*, either from the point of view of prognosis or treatment. Take, for instance, two attacks of enteric fever. One is in an athletic policeman of temperate habits. He will very likely have within a few days of the onset a nightly temperature of 104°, associated with violent delirium. The other is in a middle-aged worn-out woman, in whom the thermometer perhaps never records a higher temperature than 101°. The latter will almost certainly have a more severe attack than the former. *The extent of the pyrexia must always be read in conjunction with the resisting powers of the patient* if we are to avoid inaccuracy in prognosis or mistakes in treatment.

Signs of toxæmia we practically always get, but we do not always know the extent of the output of toxins, or even the locality of the factory where they are produced. Where we can see the site of the factory, however, we know that the output is roughly proportional to its size; but again that is a very different thing from the effect of the toxins on the patient; we want to know his resistance as well.

Let us take an example. In measles we do not at present know where the toxins are formed—probably in the circulating blood; but we do not know this for certain, and we cannot at all events estimate the output. In faucial diphtheria, on the other hand, we can see the factory, and we know from experience that the degree of toxæmia is roughly proportional to the extent of surface covered by membrane. Still we have all seen weakly children succumb to an attack in the acute stage of an attack of diphtheria where there has only been a small patch of membrane on one tonsil, because their resistance is inadequate. In dealing with the toxæmic stage of an acute specific fever we have, therefore, firstly to discover the site of the factory which will give us the extent of the output, and then to estimate the resistance of the patient.

The former is usually easy; in many diseases it is known from previous experience. Thus in diphtheria and in scarlet fever of the ordinary and septic (anginose) type the toxins are generated in the tonsils, in puerperal fever in the uterus, in erysipelas and cellulitis at the site of the local lesion, and so on. We may go wrong, however, even with many previous *post-mortem* examinations to guide us, as, for instance, in the case of enteric fever.

Here it was taught (and I am afraid the error has not yet disappeared from many of the text-books) that the toxins are generated entirely in the intestinal ulcers and perhaps in the spleen also. Now we know that the bacilli are present in the circulating blood at the very commencement of the attack, and that there are more organisms in the upper part of the intestine, especially in the duodenum, than at the site of the ulcers. Moreover, many people succumb to intense toxæmia when at the *post-mortem* examination there is found scarcely any ulceration of the intestine, and but little enlargement of the spleen. It is even probable that the intestinal ulceration is entirely secondary to the blood infection, and not *vice versa*.

Can we measure the resistance of the patient? Certainly not accurately, but we can get some help—perhaps more than is usually supposed—from the examination of the blood and from the state of the pulse. The blood count is certainly useful. The number of leucocytes varies in different diseases, but we are almost always justified in giving a bad prognosis when there is a leucopenia in the acute stage, provided that there is also evidence of a large output of toxin. Thus, a low leucocyte count in a patient suffering from enteric fever, who is obviously prostrate, is of very bad omen, and the same holds good in a case of septic scarlet fever also. The converse is not always true, but, as far as it goes, a marked leucocytosis is of favourable omen in most specific fevers, and in my experience especially so in puerperal sepsis.

The pulse is of more importance, and I could wish that nurses were more frequently taught to chart the pulse-rate as they do the temperature. This used to be the custom at Monsall Hospital, the pulse curve being marked in red ink on the temperature chart in all acute cases, and I personally found it much the more valuable of the two records. Undue rapidity of the pulse with lowering of the tension is of bad omen.

Coming now to the question of treatment, we have three main points to consider. Firstly, can we get at the site of the factory, and, if so, can we stop the supply of toxins at its source?

Obviously, *if* we can, this is the correct course to pursue, provided that the method does not unduly depress the patient.

Sometimes there is no doubt, as when we freely lay open and disinfect the site of the local lesion in cellulitis, or remove a mass of putrid placental tissue from the uterus in one form of puerperal sepsis. Sometimes we know the site but cannot disinfect it. We do not consider, for instance, that we can inject any efficient antiseptic with safety into the circulating blood in enteric fever, and I suppose that no one now holds that we can kill bacilli in an intestinal ulcer by administering antiseptics by the mouth. Also, in diphtheria (though the belief to the contrary died hard, and while it existed was responsible for the deaths of innumerable children by leading to the withholding of antitoxin), we do not think that we can help the patient by applying antiseptics to the surface of the tonsils; the active bacilli being beneath the membrane. In practice the less we do in this disease the better, on account of the danger of straining the poisoned (or paralysed) heart in the process.

The point, however, which I wish to emphasise here is the mistake which we often make by not attacking the local lesion. Witness, for instance, the futility of treating a septic endometritis in puerperal sepsis by quinine given by mouth, or a mass of necrotic temporal bone in the otitis of scarlet fever, by instructing the nurse to syringe the ear with boracic lotion. In septic scarlet fever, too, when the naso-pharynx is blocked with sloughing adenoid tissue, the removal of the putrid mass is often of undoubted advantage even though we do thereby " operate " on a patient who is suffering from fever! And in this same disease we can often do great good by repeatedly swabbing the tonsils with some strong antiseptic such as undiluted Izal, or pure peroxide of hydrogen. Certainly nothing can be more unscientific than the idea that we can adequately treat a septic local lesion entirely by medicines given internally.

But what about the general treatment of the toxæmia itself? Obviously, we neutralise the toxins directly if we can, but the only specific fever in which this is at present possible is diphtheria, so we need not consider this further. Nor are drugs, such as quinine or salicylate of soda (except, of course, in malaria or acute rheumatism), of much value, and they often, especially the latter, do a good deal of harm.

What we can do, however, is to assist in their elimination from the body, and with this in view I would lay great stress on the importance of administering large quantities of fluid. Outside fever hospitals the belief is rampant that a feverish child should not drink when it is thirsty, especially of cold water. Can there be any better remedy? I think not. In private practice, however, one is often hampered greatly in this respect by the interference of elderly relatives, and it is greatly to be wished that the popular prejudice would die out. The same applies to warm or cool baths, which are of undoubted service. When the patient is too ill to drink, massive saline injections given subcutaneously and slowly are often invaluable. Personally, I much prefer this method to the intravenous route, and there is not the same danger of causing œdema of the lungs if the quantity of saline given be inadvertently too large. Continuous irrigation *per rectum* is sometimes useful in adults, but it requires the constant attendance of a skilled nurse, and is unsuitable for children.

Lastly, it is necessary to encourage the formation of antitoxins by the patient's own leucocytes, by such measures as skilled and frequent feeding, and in some cases by the judicious use of stimulants. More directly we can sometimes usefully produce a leucocytosis by the use of nucleins, and in practice, especially in children, Virol is of service here. I have not found the subcutaneous injection of nucleic-acid solutions of much practical benefit however. Whenever possible it is better to nurse toxic cases in the open air, and it is desirable that provision for this should be made by architects in designing fever hospitals.

